# First-in-human study of FLT3 CAR-T cell therapy for relapsed acute myeloid leukemia

**DOI:** 10.1038/s41698-026-01466-2

**Published:** 2026-05-14

**Authors:** Xiaojin Wu, Gao Lu, Lei Tian, Suning Chen, Michael A. Caligiuri, Jianhua Yu

**Affiliations:** 1 The First Affiliated Hospital of Soochow University, Suzhou, China; 2 Division of Hematology & Oncology, Department of Medicine, School of Medicine, University of California, Irvine, CA USA; 3 Department of Hematology and Hematopoietic Cell Transplantation, and the Hematologic Malignancies Research Institute, City of Hope National Medical Center, Los Angeles, CA USA; 4 Clemons Family Center for Transformative Cancer Research, Chao Family Comprehensive Cancer Center, University of California, Irvine, CA USA

**Keywords:** Cancer, Oncology, Stem cells

## Abstract

In this first-in-human open-label study, autologous FLT3 CAR-T cells were delivered to two patients with relapsed and refractory FLT3^+^ AML. Bone marrow examination revealed AML blasts with high but variable surface density of FLT3 expression. Following tumor cytoreduction and lymphodepletion, 1 × 10^6^/kg of FLT3 CAR-T cells were administered, resulting in in vivo CAR-T cell expansion and grade 1 cytokine release syndrome in both patients. Both patients failed to achieve remission after CAR-T cell therapy, but bone marrow examination following therapy revealed the elimination of FLT3^+^ AML blasts, persistence of FLT3 ^−^ AML blasts, and early post-treatment preservation of normal CD34^+^ hematopoietic stem and progenitor cells (HSPCs) with variable FLT3 expression. Collectively, autologous FLT3 CAR-T cells can be safely administered and can eradicate FLT3⁺ blasts with minimal toxicity, without causing substantial damage to normal CD34⁺ HSPCs; however, they fail to induce leukemic remission due to the heterogeneity of FLT3 expression and the persistence of FLT3⁻ AML blasts.

## Introduction

Acute myeloid leukemia (AML) is characterized by high heterogeneity at both the cellular and molecular levels^1^. Accumulation of mutations during leukemogenesis leads to a diverse population of leukemia cells^2^. Though patients with low or average risk AML could prolong their survival with standard chemotherapy, most still progress to relapsed or refractory (R/R) AML, especially in the elderly, where the disease is prevalent. In recent years, in addition to hematopoietic stem cell transplantation (HSCT), immunotherapy using chimeric antigen receptor (CAR)-T cells targeting CD33^3^, CLL-1^4^, or CD123^5^ has emerged as a treatment option for these R/R AML patients. However, 3 of 3 AML patients treated with CD33 CAR-T cells experienced leukopenia on the day of infusion, and none achieved a response^3^. In a CLL-1 CAR-T cell trial, among 10 enrolled patients, 7 achieved complete response (CR) or CR with incomplete hematologic recovery (CRi); however, all developed cytokine release syndrome (CRS)—4 low-grade and 6 high-grade—and 7 developed severe pancytopenia^4^. Most recently, 3 of 12 AML patients treated with CD123 CAR T cells achieved a response; however, the therapy overall is associated with high rates of cytokine release syndrome and relatively poor clinical efficacy^5^.

FMS-like receptor tyrosine kinase 3 (FLT3) comprises an intrinsic tyrosine kinase domain, a transmembrane domain, a juxtamembrane domain, and an extracellular receptor. By binding to its FLT3 ligand (FL), FLT3 activates the PI3K/AKT or RAS/MAPK signaling pathway, thereby regulating proliferation, survival, and differentiation of early hematopoietic stem and progenitor cells (HSPCs)^6^. This protein is frequently expressed on AML blasts but at low levels on most CD34^+^ HSPCs and their progeny^7,8^, suggesting that FLT3 is a reasonable target for CAR-T cell-based immunotherapy. We first generated FLT3 CAR-T cells and demonstrated their efficacy and safety in humanized mouse models of AML^9^, while others have also shown that targeting FLT3 may be toxic to HSPCs^10^.

In this report, we describe the first two patients diagnosed with R/R FLT3^+^ AML who received FLT3 CAR-T cell therapy in a clinical trial. Autologous T cells were isolated from the apheresis product and transduced with the LVV-CI-CAR-FLT3 lentiviral vector containing an anti-human FLT3 single-chain variable fragment (scFv), along with a CD28 costimulatory domain and a CD3ζ activation signaling domain. FLT3 CAR-T cells were manufactured under good manufacturing practice (GMP) conditions using a method modified from the previously described protocol^9^. We administered FLT3 CAR-T cells intravenously to the first two patients diagnosed with R/R FLT3^+^ AML.

## Results

### FLT3 CAR-T therapy eradicate FLT3^+^ blasts in relapsed AML patients

**Fig. 1:**
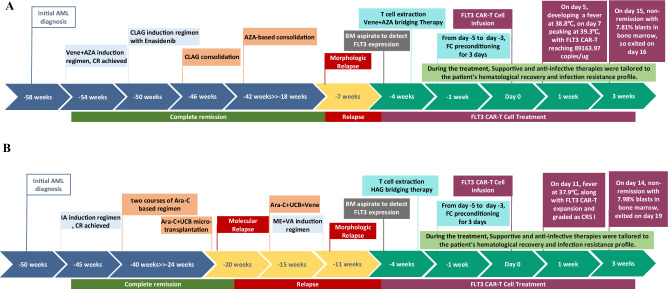
Clinical timeline of two patients with acute myeloid leukemia (AML). Clinical course of patients 1 (**A**) and 2 (**B**) with AML. AML acute myeloid leukemia, CR complete remission, Vene venetoclax, AZA azacitidine, CLAG cladribine plus low-dose Ara-C (cytarabine) and G-CSF, Ara-C cytarabine, IA idarubicin plus cytarabine, FC fludarabine plus cyclophosphamide, ME mitoxantrone plus etoposide, VA venetoclax plus azacitidine, UCB umbilical cord blood, HAG homoharringtonine plus cytarabine and G-CSF.

**Fig. 2:**
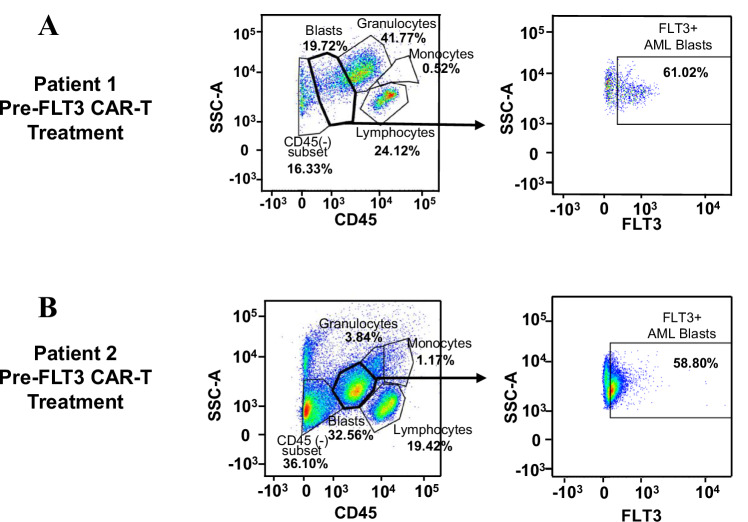
FLT3 surface expression on blasts in bone marrow in the two enrolled patients. **A** Patient 1 prior to FLT3 CAR-T cell therapy had 19.72% AML blasts among total nucleated bone marrow cells, of which 61.02% expressed FLT3, as assessed by flow cytometry. **B** Patient 2 prior to FLT3 CAR-T cell therapy had 32.56% AML blasts among total nucleated bone marrow cells, of which 58.80% expressed FLT3, as assessed by flow cytometry.

**Fig. 3:**
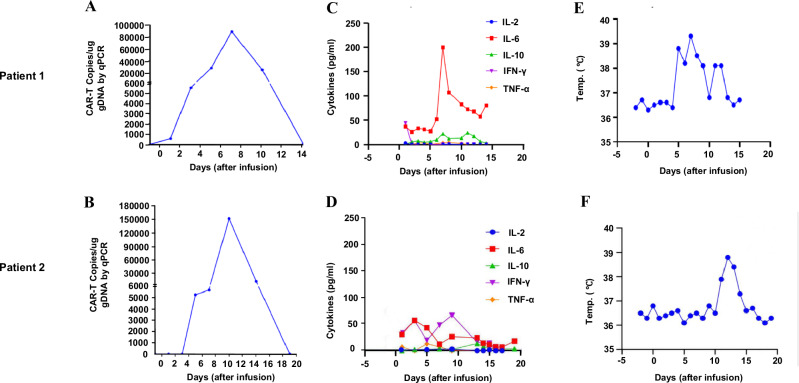
The dynamic changes of FLT3 CAR-T cells, serum cytokine levels, and body temperatures in patients 1. **A**, **B** cDNA quantification of FLT3 CAR-T cells in patents 1 (**A**) and 2 (**B**). **C**, **D** Serum cytokine levels in patients 1 (**C**) and 2 (**D**). **E**, **F** Body temperature in patients 1 (**E**) and 2 (**F**). Day 0 was the day when FLT3 CAR-T cells were infused in to the patients.

Patient 1, a 60-year-old woman, was diagnosed with AML, identified with NPM1 and IDH2 mutations, without mutations in FLT3. Patient 2, a 20-year-old woman, was diagnosed with AML with a karyotype of 46, X, dic(X;13)(p23;q13), t(8;21)(q22;q22), along with CSF3R and ZBTB7A mutations, and no FLT3 mutations. According to the European Leukemia Net (ELN) 2022 risk stratification^11^, this patient was classified as having a favorable risk profile. Figure 1A shows the treatment schedules of the two patients. Following salvage chemotherapy, patient 1 was verified to progress to a morphological relapse with 19.72% myeloid blasts in the bone marrow, of which 61.02% expressed FLT3 (Fig. 2A), and patient 2 was verified to progress to a morphological relapse with 32.56% myeloid blasts, of which 58.80% expressed FLT3 (Fig. 2B). Both patients subsequently consented to autologous FLT3 CAR-T therapy due to the high FLT3 expression on leukemic blasts at relapse. The two patients each received bridging chemotherapy, as noted in Fig. 1, followed by a 3-day fludarabine and cyclophosphamide regimen for lymphodepletion. Two days after lymphodepletion, autologous FLT3 CAR-T cells were infused as a single dose of 1 × 10^6^/kg (day 0). Copies of FLT3 CAR-T cells measured by quantitative real-time PCR (qPCR) were noted to increase after administration, reaching a peak on day 7 (89,163.97 copies/μg) in patient 1 (Fig. 3A) and on day 10 in patient 2 ( > 150,000 copies/μg) (Fig. 3B). Both patients experienced grade I cytokine release syndrome (Fig. 3C-F). Blood counts improved following the transfusion of platelets and red blood cells after the resolution of CRS. No other significant adverse events were observed during the treatment.

**Fig. 4:**
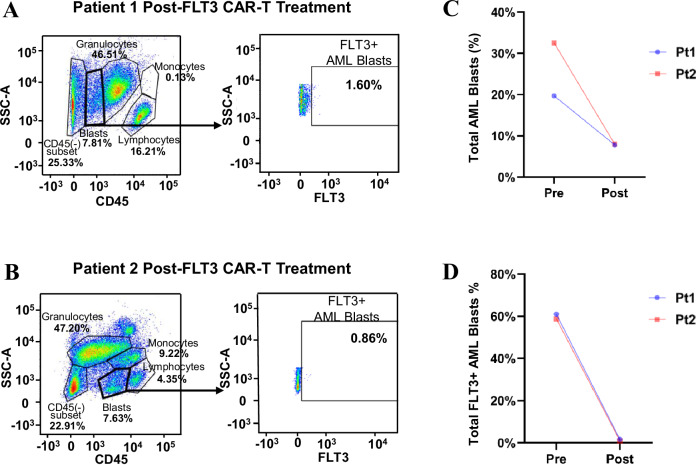
Reduction in AML blasts following FLT3 CAR-T cell therapy. **A** Following FLT3 CAR-T cell therapy, patient 1 had 7.81% AML blasts among total nucleated bone marrow cells, of which 1.60% expressed FLT3. **B** Following FLT3 CAR-T cell therapy, patient 2 had 7.63% AML blasts among total nucleated bone marrow cells, of which 0.86% expressed FLT3. **C** Percentage change in total AML blasts among nucleated bone marrow cells prior to and after FLT3 CAR-T cell therapy. **D** Percentage change in FLT3⁺ AML blasts among total blasts prior to and after FLT3 CAR-T cell therapy.

Flow cytometric analysis of bone marrow aspirates on day 15 following FLT3 CAR-T cell infusion in patient 1 shows a reduction of AML blasts from 19.72%, of which 61.02% express FLT3 (Fig. 2A), to 7.81% blasts, of which only 1.60% express FLT3, i.e., 98.40% of the remaining AML blasts lack FLT3 expression (Fig. 4A). Likewise, flow cytometric analysis of bone marrow aspirates on day 14 following FLT3 CAR-T cell infusion in patient 2 shows a reduction of AML blasts from 32.56%, of which 58.80% express FLT3 (Fig. 2B), to 7.63% blasts, of which only 0.86% express FLT3, i.e., 99.14% of the remaining AML blasts lack FLT3 expression (Fig. 4B). The reduction in total blasts is illustrated for both patients in Fig. 4C, while the profound reduction in total FLT3^+^ AML blasts is illustrated for both patients in Fig. 4D. The participants exited the trial on day 16 (patient 1) and on day 19 (patient 2) because of AML relapse. The immunophenotype of the AML blasts remained unchanged throughout their palliative care.

### CD34⁺ HSPCs appear resistant to FLT3 CAR-T cell therapy in AML patients

**Fig. 5:**
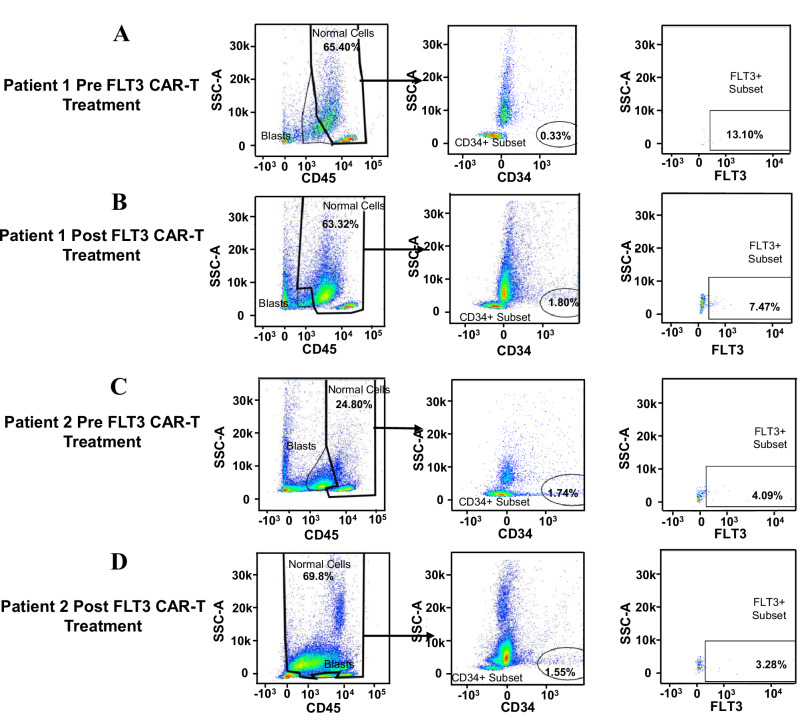
Normal HSPCs are resistant to FLT3 CAR-T cell–mediated depletion. FLT3 expression on normal CD34⁺ hematopoietic stem cells assessed by flow cytometry before FLT3 CAR-T cell therapy in patient 1 (**A**) and patient 2 (**C**), and after FLT3 CAR-T cell therapy in patient 1 (**B**) and patient 2 (**D**).

We performed flow cytometric analyses of G-CSF–mobilized CD34⁺ HSPCs from healthy donors and AML patients with no CAR-T treatment (Supplementary Fig. 1), as well as normal CD34⁺ HSPCs in the bone marrow prior to and after FLT3 CAR-T cell infusion in patients 1 and 2 (Fig. 5). Compared with the low surface FLT3 expression levels on normal CD34⁺ HSPCs from healthy donors and AML patients (Supplementary Fig. 1A–D), leukemia blasts from de novo AML presented increased FLT3 expression (Supplementary Fig. 1E, F). After FLT3 CAR-T treatment, normal CD34⁺ HSPCs from patients 1 and 2 showed low but relatively sustained FLT3 expression despite its disappearance on malignant AML blasts, suggesting that normal HSPCs in the two patients may be resistant to FLT3 CAR-T cell–mediated depletion, at least during the patients’ short follow-up period (Fig. 5A–D).

## Discussion

FLT3 CAR-T cell infusions at a dose of 1 × 10^6^ cells/kg in the two cases reported here were delivered safely and were well tolerated, without significant adverse effects. In our study, expansion of autologous FLT3 CAR-T cells in both patients with FLT3⁺ AML, together with virtual elimination of FLT3⁺ AML blasts and the development of low-grade CRS, indicates in vivo immunologic activity of these effector cells against the target antigen. Both patients presented with multiple relapsed/refractory AML, and the majority of AML blasts in both patients exhibited high surface density expression of FLT3 prior to FLT3 CAR-T cell infusion. Following the infusion of FLT3 CAR-T cells, FLT3 expression was virtually absent on residual AML blasts approximately two weeks after the infusion. Thus, while the effector cell eliminates its target cell population, the emergence of exclusively FLT3⁻ clonal or oligoclonal AML blasts at relapse highlights the limitations of single-antigen CAR T cell therapy in AML^12–17^. We demonstrated a successful on-tumor-on-target effect of the FLT3 CAR T therapy in both AML patients, as we had previously shown in our preclinical model of FLT3 AML^9,18^. The relapse of both patients with AML blasts having very low-to-absent FLT3 expression following FLT3 CAR-T cell therapy also strongly suggests that FLT3 CAR-T therapy alone is likely to be ineffective in achieving a complete remission in the vast majority of patients with FLT3^+^ AML. As with FLT3 expression on each patient’s AML blasts in this study, its expression on normal CD34^+^ HSPCs is not homogeneous^19^. Although a small population of normal CD34^+^ HSPCs was detected in AML bone marrow—consistent with a previous report^20^, we observed relatively weak FLT3 expression on these cells compared with AML blasts, a finding also supported by other studies^7,8^. Notably, it appears that normal HSPCs were spared from the cytolytic effects of FLT3 CAR-T infusion in both patients and, unlike the residual AML blasts, exhibited some FLT3 expression post the CAR-T cell treatemnt, consistent with a minimal on-target, off-tumor effect, as we previously observed in our mouse model^9^.

The two patients enrolled in our study had FLT3 surface expression on only a subset (~60%) of their AML blasts, leaving the remaining (~40%) untargeted and therefore potentially invisible to our FLT3-targeted therapy. As seen in chemotherapy-resistant AML, different clusters of leukemia cells respond differently to therapies^21^. Patterns of clonal evolution under therapy-related pressure may give rise to transient shifts in the dominant phenotypes of leukemia cells^22^. What is interesting in this case is that, even though each patient had developed strong resistance to chemotherapy over the short course of their disease, the FLT3^+^ AML in each case remained highly sensitive to T-cell-mediated therapy. Thus, if the equivalent of CD19 expression in human B cells—that is, a ubiquitously expressed tumor-associated antigen—were identified in AML, AML blasts, and therefore patients, they could be susceptible to CAR-T cell therapy even after failed chemotherapy and allogeneic stem cell transplantation. However, such uniformly expressed tumor-associated antigens remain elusive and require identification and clinical confirmation in AML. Under these circumstances, targeting multiple tumor-associated myeloid antigens with a combination of CAR-T cells directed against these antigens may represent an optimal strategy for achieving sustained complete remission in AML—an outcome that has thus far proven elusive with more conventional CAR-T therapies such as those targeting CD33^3^ and CLL-1^4^. Although we performed the study in just two patients, FLT3 CAR-T cells, compared with CD33 CAR-T cells^23,24^ and CLL-1 CAR-T cells^4,25^, demonstrated robust in vivo expansion in both treated patients and were associated with manageable on-target, off-tumor toxicity. Notably, no grade ≥ 3 cytokine release syndrome (CRS) was observed with FLT3 CAR-T, whereas reported rates of grade ≥ 3 CRS were 33% in CD33 CAR-T trials and 60% in CLL-1 CAR-T studies. In addition, FLT3 CAR-T effectively eliminated FLT3⁺ AML blasts.

Our study, however, has several limitations. First, our results can only be considered preliminary because only two patients were evaluated. Second, due to the widespread clinical use of FLT3 inhibitors such as gilteritinib, enrollment of patients with relapsed/refractory FLT3-ITD–positive AML was challenging. The resulting selection of FLT3-ITD–negative patients precluded direct evaluation of FLT3 CAR-T efficacy in FLT3-ITD–positive AML, despite the CAR being designed to target surface FLT3 expression irrespective of mutation status. Third, early patient withdrawals from the trial resulted in relatively short follow-up, preventing assessment of long-term outcomes. Fourth, while our data suggest in vivo targeting selectivity between FLT3⁺ blasts and CD34⁺ HSPCs in the short term, the long-term hematopoietic safety and functional integrity of the HSPC compartment after FLT3 CAR-T therapy require evaluation in future studies with longer follow-up. Fifth, as others have done, we performed bone marrow aspirates to quantify blasts prior to bridging therapy and lymphodepletion; therefore, the reduction of FLT3⁺ blasts could partially reflect the effect of bridging therapy and lymphodepletion. However, this is unlikely to be a major factor, as the pre-CAR-T regimen would not be expected to selectively reduce FLT3⁺ blasts. Instead, post our CAR-T therapy, we observed FLT3⁻ blasts emerged, whereas FLT3⁺ blasts did not. Finally, the lack of genetic, transcriptional, or epigenetic analyses post–CAR-T infusion limits our ability to determine the origin of the emergent FLT3⁻ blasts, specifically whether they arose from pre-existing clones or through antigen downregulation.

In summary, we have demonstrated that autologous FLT3 CAR-T cells can be administered safely without significant (grade 3 or 4) adverse events to two patients with FLT3^+^ AML. We observed the elimination of FLT3^+^ blasts in both patients following treatment, alongside the emergence of FLT3^–^ AML blasts. This phenomenon could be potentially explained by heterogeneous antigen expression and/or antigen loss. Interestingly, normal CD34⁺ HSPCs showed no substantial changes between pre- and post-therapy samples, indicating a potential resistance of normal HSPCs to FLT3 CAR-T cell therapy. Given the heterogeneity of tumor-associated antigen expression in most, if not all, AML cases, a combination of CAR-T cell therapies targeting multiple tumor-associated myeloid antigens will likely be necessary for effective elimination of AML. While this limited first-in-human study demonstrates feasibility and preliminary biological activity, the findings require validation in larger prospective cohorts. More innovative strategies, based on mechanistic insights, should be considered to improve CAR-T cell efficacy in patients with AML.

## Methods

### Manufacture of CAR-T product and treatment

The preclinical studies of FLT3 CAR T cells were described previously^9,26^. Peripheral blood mononuclear cells were collected by apheresis from enrolled patients. Autologous T cells were isolated from the apheresis product and transduced with the LVV-CI-CAR-FLT3 lentiviral vector containing an anti-human FLT3 scFv, along with a CD28 costimulatory domain and a CD3ζ activation signaling domain. FLT3 CAR-T cells were manufactured under GMP conditions. The manufacturing process involved T cell isolation, activation, lentivirus transfection, T cell expansion and culturing, transfection rate detection, cellular quality control measures, cell infusion procedures, and post-infusion monitoring. The manufactured FLT3 CAR-T cells were formulated and were infused in a volume of 10–20 ml within 2 h after thawing.

### Study design

Considering the anti-leukemia effect and safety of FLT3 CAR-T cells in the preclinical stage, we initiated this single-arm and open-label clinical trial (NCT06760260, registration date 12/30/2024) to investigate the feasibility and safety of autologous FLT3 CAR-T cells. The primary objective of this first-in-human study was to test the safety of our product, focusing on an initial assessment of possible on-target/off-tumor toxicity against FLT3-expressing normal HSPCs in patients with AML whose alternative therapeutic options were exhausted. Patients were selected based on FLT3 membrane expression by flow cytometry, irrespective of FLT3-ITD mutation status. To facilitate comparison, cell counts are normalized across different mean fluorescence intensity (MFI) values, as previously reported^8,27^. This study was conducted in accordance with the ethical principles of the Declaration of Helsinki and approved by the Ethics Committee of the First Affiliated Hospital of Soochow University. Adults aged 18–70 years with primary or secondary AML who were refractory to three courses of standard induction therapy or relapsed after achieving CR, and who were either FLT3 mutation–positive by genetic testing or had FLT3 expression on ≥ 35% of bone marrow blasts, were eligible. The two enrolled patients met these requirements and provided written informed consent to participate.

### Patient treatment

Both patients had relapsed AML at the time of peripheral blood mononuclear cell (PBMC) apheresis for CAR-T cell preparation (Fig. 1). After apheresis, patients received bridging chemotherapy followed by lymphodepleting preconditioning with fludarabine (30 mg/m^2^/day on days −5 to −3) and cyclophosphamide (300 mg/m²/day on days −5 to −3). Two days after lymphodepletion, each patient received a single infusion of autologous 1 × 10^6^ FLT3 CAR-T cells/kg (day 0, D0). Bone marrow aspirates were conducted to detect FLT3 expression on blasts and normal HSPCs prior to the bridging chemotherapy and after the CAR-T treatment. CAR-T cell proliferation was analyzed using quantitative real-time PCR (qPCR) on days 1, 3, and 5, and at subsequent time points. Cytokines are measured by enzyme-linked immunosorbent assay (ELISA) during CAR-T treatment. Patients were monitored for adverse effects throughout the study, which were graded according to the Common Terminology Criteria for Adverse Events, version 5.0 (NCI-CTCAE v5.0). Infection prophylaxis after CAR-T cell infusion was administered individually, as needed, in accordance with institutional standards.

## Supplementary information

Supplemental Figure 1

## Data Availability

Raw data can be requested from the corresponding authors for academic use, subject to approval of a research plan, a data transfer agreement, and ethics committee approval.
