# Supplementary material for: First-in-human study of FLT3 CAR-T cell therapy for relapsed acute myeloid leukemia
Source: NPJ Precis Oncol. 2026 May 14;10:291. doi: 10.1038/s41698-026-01466-2 (PMC13402607; doi:10.1038/s41698-026-01466-2)
Supplement: Supplementary file 1 — Supplemental Figure 1 [file 41698_2026_1466_MOESM1_ESM.pdf]

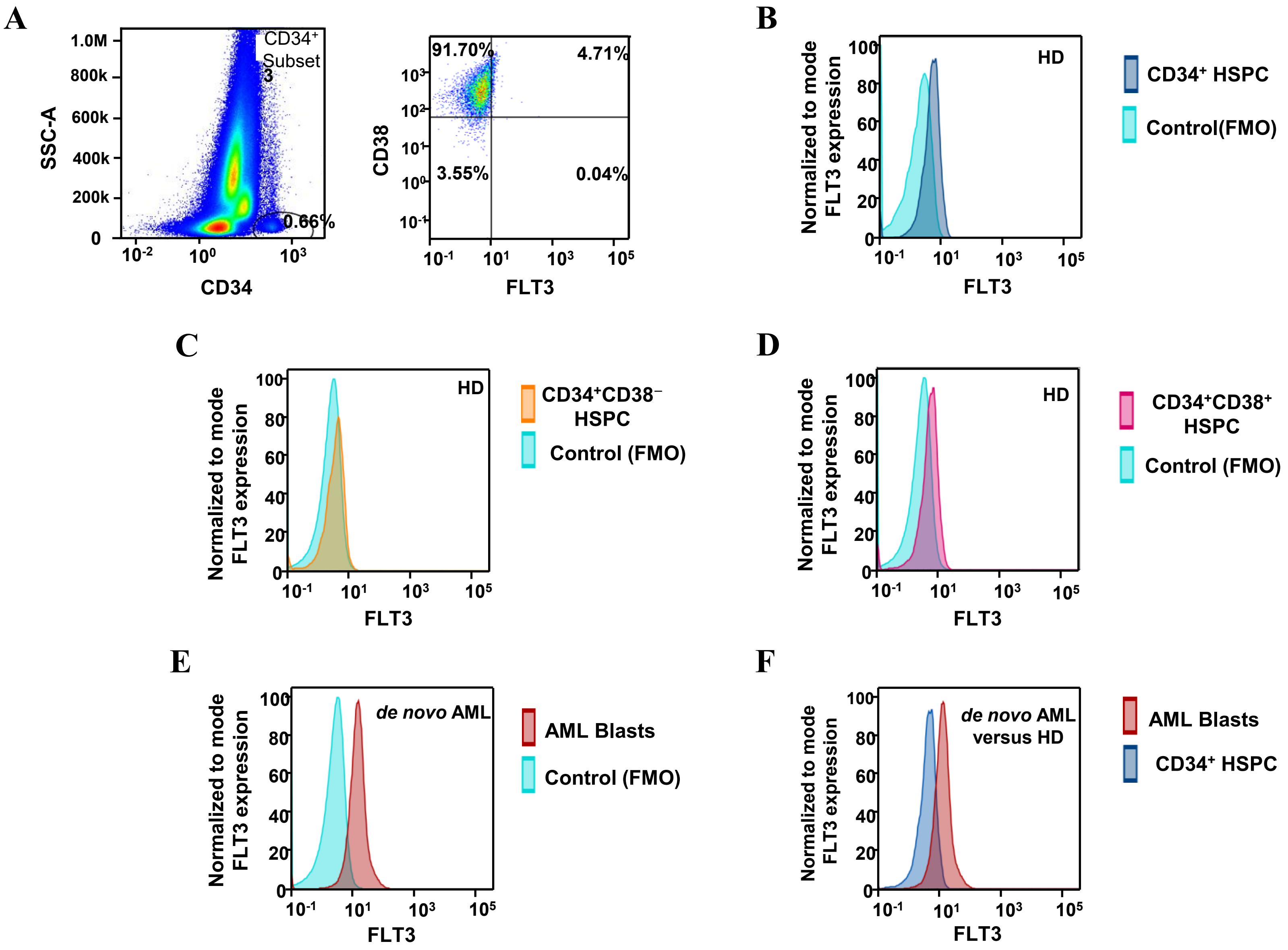

Supplementary Figure 1 | FLT3 surface expression on CD34+ HSPCs from healthy donors and AML blasts. (A, B) Surface FLT3 expression of CD34+ healthy donor (HD) HSPCs was assessed by flow cytometry. (C, D) Surface FLT3 expression of CD34+CD38− (C) and CD34+CD38+ (D) HD HSPCs, assessed by flow cytometry. (E, F) Surface FLT3 expression on de novo AML blasts (E) and its comparison with normal CD34+ HSPCs from the same AML patient (F). AML, acute myeloid leukemia; HSPC, hematopoietic stem and progenitor cells. Cell counts are normalized across different mean fluorescence intensity (MFI) values. Representative data from one of seven healthy donors and one of four AML patients are shown.
